# Occurrence of Hand-Foot-and-Mouth Disease Pathogens in Domestic Sewage and Secondary Effluent in Xi’an, China

**DOI:** 10.1264/jsme2.ME11352

**Published:** 2012-03-23

**Authors:** Zheng Ji, Xiaochang Wang, Chongmiao Zhang, Takayuki Miura, Daisuke Sano, Naoyuki Funamizu, Satoshi Okabe

**Affiliations:** 1 Key Laboratory of Northwest Water Resource, Ecology and Environment, Ministry of Education, Xi’an University of Architecture and Technology, Xi’an, Shaanxi, 710055, China; 2 Division of Environmental Engineering, Faculty of Engineering, Hokkaido University, North 13, West 8, Kita-ku, Sapporo, Hokkaido, 060–8628, Japan

**Keywords:** *Enterovirus*, hand-foot-and-mouth disease, phylogenetic analysis, semi-nested RT-PCR, wastewater

## Abstract

Hand, foot and mouth disease (HFMD), caused by a group of enteric viruses such as *Enterovirus 71* (EV71), *Coxsackievirus A16* (CVA16) and *Coxsackievirus A10* (CVA10), is heavily epidemic in East Asia. This research focused on investigating the occurrence of HFMD pathogens in domestic sewage and secondary effluent before disinfection in a wastewater treatment plant (WWTP) in Xi’an, the largest megacity in northwest China. In order to simultaneously detect all three HFMD pathogens, a semi-nested RT-PCR assay was constructed with a newly designed primer set targeting conservative gene regions from the 5′ untranslated region (UTR) to VP2. As a result, 86% of raw sewage samples and 29% of the secondary effluent samples were positive for the HFMD viral gene, indicating that HFMD pathogens were highly prevalent in domestic wastewater and that they could also persist, even with lower probability, in the secondary effluent before disinfection. Of the three HFMD pathogens, CVA10 was positive in 48% of the total samples, while the occurrences of CVA16 and EV71 were 12% and 2%, respectively. It could thus be stated that CVA10 is the main HFMD pathogen prevailing in the study area, at least during the investigation period. High genetic diversity in the conservative gene region among the same serotype of the HFMD pathogen was identified by phylogenetic analysis, implying that this HFMD pathogen replicates frequently among the population excreting the domestic sewage.

*Human enterovirus* (HEV), a genus of positive-sense single-stranded RNA virus, is associated with a number of clinical infections (14). Most cases of enterovirus infection are asymptomatic or mild, and can usually recover without any special medication (24). Hand, foot and mouth disease (HFMD) is an acute enterovirus infection, characterized by a brief febrile illness and vesicular lesions on the hands, feet, mouths and buttocks of infected people (22). In recent years, numerous large outbreaks of HFMD have occurred in the Asia-Pacific region, including China (29, 30), Singapore (1, 21, 25), South Korea (10), Malaysia (3), Vietnam (22), and Japan (6, 8).

*Enterovirus 71* (EV71), *Coxsackievirus A16* (CVA16) and *Coxsackievirus A10* (CVA10), members of *Human enterovirus A* (HEV-A) species, are major etiologic agents of HFMD (15), and have caused significantly high morbidity and mortality in China (27, 28). Enteroviruses are excreted from infected individuals with feces for up to 11 weeks (4), thereby possibly existing in domestic wastewater at high titers. Since enteric viruses are not removed efficiently by conventional wastewater treatment processes, and viral contamination of water environment may occur due to the discharge of effluent that contains human pathogenic viruses if proper disinfection is not performed (19, 23). Although the contamination level of environmental waters with various pathogenic viruses including EV71 has been revealed (2, 7, 9, 12, 20), few studies have been conducted by far for a comparison of the occurrences of different HFMD viral pathogens in the water environment.

This study thus aims to establish a general molecular biological method with a newly designed primer set for detecting and comparing the occurrence of EV71, CAV10, and CAV16, the three main HFMD viral pathogens. The method was then applied to monitor the occurrence of these HFMD viral pathogens in a domestic wastewater treatment plant (WWTP) in Xi’an, the largest megacity in northwest China, in a seven-month period. The extent of HFMD pathogen occurrence was evaluated by the frequency of positive samples with HFMD genes from domestic sewage and secondary effluent before disinfection. The main HFMD pathogens were identified by gene sequence determination, and phylogenetic analysis of the acquired gene was performed to elucidate the genetic diversity of HFMD pathogens in the investigation area.

## Materials and Methods

### Design of specific primers

A primer set was designed based on the conservative gene regions of whole genome sequences of CAV10, CAV16 and EV71 to detect these HFMD viral pathogens simultaneously. The complete nucleotide sequences of five EV71 strains, five CVA16 strains and one CVA10 strain were retrieved from the National Center for Biotechnology Information (NCBI) database, GenBank, and multiple alignment was created with Clustal X 1.83 to design a primer set with its feasibility checked using Primer3 (v. 0.4.0) (http://frodo.wi.mit.edu/primer3/).

### Water sample processing

The constructed method was applied to investigate the occurrence of HFMD pathogens in a WWTP in Xi’an, China where a conventional activated sludge process is applied for treating domestic wastewater with a capacity about 150,000 m^3^/d. Water sampling was conducted for about 7 months from November 2010 to May 2011 at 10-day intervals so that 21 batches were collected, each including the raw sewage and secondary effluent before disinfection by chlorine. The sampling period almost covered the spring season (from March to May) when HFMD was prevailing in general (28).

Polyethylene glycol (PEG) precipitation was employed to recover viruses from wastewater samples according to Lewis and Metcalf (13) with some modifications. PEG6000 and sodium chloride were added to yield the final concentrations of 8% (w/v) and 2.3% (w/v), respectively. The mixture was stirred gently at about 80 rpm, incubated at 4°C overnight and then centrifuged at 9,000×*g* for 30 min at 4°C. The supernatant was discarded and the pellet was suspended in 1 mL deionized distilled water (DDW) with a vortex mixer.

### RNA extraction and semi-nested RT-PCR amplification

Total viral RNA was extracted from 140 μL of 1 mL virus concentrate using the QIAamp Viral RNA Mini Kit (Qiagen, Valencia, CA, US). According to the spin protocol described by the manufacturer, 60 μL viral RNA was obtained. Complementary DNA (cDNA) was synthesized from 2 μL of 60 μL extracted RNA with DNase treatment, using the PrimeScript RT Reagent Kit with gDNA Eraser (Takara, Otsu, Japan) according to the protocol described by the manufacturer. Semi-nested PCR was employed to amplify the viral gene extracted from the wastewater samples. First-round PCR was carried out using the synthesized cDNA as a template. The reaction mixture consisted of 2 μL cDNA, 0.25 μL *Ex Taq* (Takara), 2.5 μL of 10×*Ex Taq* Buffer, 2 μL dNTP Mixture, and 400 nM concentration of each 1st-round PCR primer, all mixed with DDW to a total volume of 25 μL. The template of the 2nd-round PCR was 1 μL of the 100-fold diluted product from the 1st PCR. Each reaction was performed under the following conditions: initial denaturation at 95°C for 5 min, 35 cycles of amplification with denaturation at 95°C for 30 s, annealing at 56°C for 30 sec, extension at 72°C for 1 min, and an additional 4 min for elongation in the final cycle. The PCR products were electrophoresed in 1.5% (w/v) agarose gel, stained with GelRed Nucleic Acid gel stain (Biotium, Hayward, US), and visualized by UV illumination.

### Specificity and sensitivity tests

The specificity and sensitivity of semi-nested RT-PCR were tested using the target viruses (CVA10, CVA16 and EV71) and a non-target virus, *Coxsackievirus B4* (CVB4) supplied by the National Institute of Infectious Diseases, Japan, and another non-target virus, Poliovirus type 1 (PV-1/Sabin strain) supplied by Tokyo Metropolitan Institute of Public Health, Japan. Viral cDNA from target and non-target viruses were quantified according to Monpoecho *et al.*(16), and 10^5^ copies of each viral cDNA were applied to semi-nested RT-PCR in the specificity test. Ten-fold serial dilutions of the viral cDNA of target viruses were applied to semi-nested RT-PCR in the sensitivity test. All PCR products were visualized as described above.

### Sequencing and phylogenetic analysis

The purified amplicons were used for the sequencing reaction to identify the sequences originating from HFMD viral pathogens. The semi-nested PCR products were gel-isolated and purified using the Bioteke agarose gel purification kit (Bioteke, Beijing, China) following the manufacturer’s instructions. The purified DNA was sent to Sangon Biotech (Shanghai, China) for sequence determination. When multiple sequences were observed in a single sample, the semi-nested PCR products were cloned into a pMD19-T Simple Vector (Takara) and five clones from each sample were sequenced. The raw sequence data were analyzed with Chromas software (version 2.31) to obtain the final sequence to compare with those published in the NCBI database using the Basic Local Alignment Search Tool (BLAST) program. The phylogenetic tree of the acquired viral genes was constructed using Clustal X 1.83, and depicted with Njplot (http://pbil.univ-lyon1.fr/software/njplot.html).

### Nucleotide sequence accession numbers

The nucleotide sequences of the semi-nested PCR products acquired in this study have been deposited in the Genbank database under accession numbers JN086565 to JN086604.

## Results and Discussion

### Primer design

**Fig. 1:**
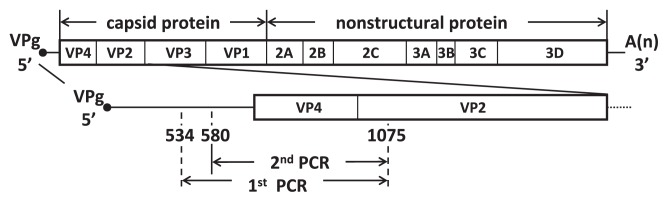
Genome structure of EV71 (accession number: EU703812) and primer locations.

A primer set for semi-nested RT-PCR was designed in relatively highly conserved regions among the three HFMD viruses. The appropriate sites for simultaneous amplification of the three HFMD viral pathogens were observed both in their 5′ untranslated region and capsid protein-coding regions (from VP4 to VP2) (Fig. 1). The primer sequences are: 546F (1st round forward primer), 5′-CGGAACCGACTACTTTGG-3′; 592F (2nd round forward primer), 5′-TGGCTGCTTATGGTGACA-3′; 1090R (reverse primer), 5′-GCARTASKMRGGCCAYTC-3′.

**Fig. 2:**
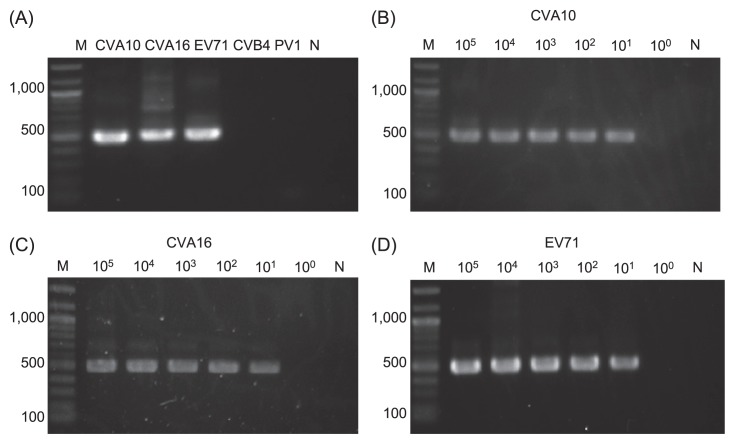
Specificity and sensitivity tests of semi-nested PCR. (A) Reaction specificity to viral cDNA (10^5^ copies/1st PCR reaction). (B), (C) and (D) Reaction sensitivity to viral cDNA of CVA10, CVA16 and EV71, respectively. M, marker; N, negative control.

The reaction specificity of semi-nested RT-PCR was tested for viral cDNA of the three target viruses (CVA10, CVA16 and EV71) and two non-target viruses (CVB4 and PV1). As can be seen from Fig. 2A, clear bands were observed for the target viruses while no products were obtained for the non-target viruses, demonstrating that semi-nested RT-PCR specifically targets HFMD viral pathogens. The reaction sensitivity was also analyzed by using ten-fold serial dilutions of viral cDNA. As can be seen from Fig. 2B, 2C and 2D, clear bands could be obtained for all the three HFMD viral pathogens from 10^5^ to 10^1^ copies/1st PCR reaction, indicating that semi-nested RT-PCR is equally sensitive to these viruses. The detection limit of semi-nested RT-PCR could thus be confirmed as 10^1^ copies/1st PCR reaction.

### Detection of HFMD viral pathogens in wastewater samples from a WWTP

**Fig. 3:**
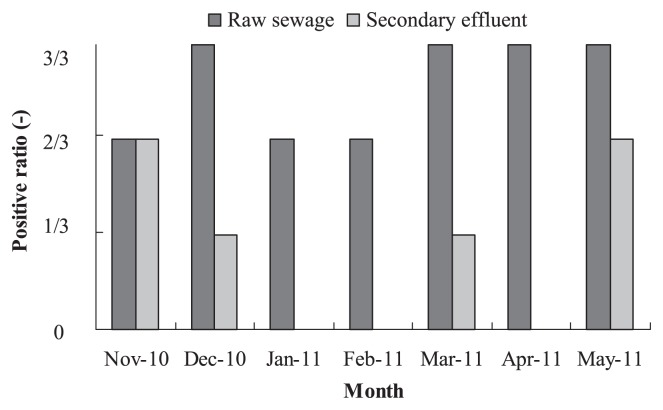
Positive ratio of HFMD pathogens in raw sewage and secondary effluent samples from a WWTP in Xi’an, China.

As a result of semi-nested RT-PCR analysis, 86% (18 of 21) of raw sewage samples and 29% (6 out of 21) of secondary effluent samples were found to be positive for the HFMD viral gene, indicating that HFMD pathogens were prevalent in domestic wastewater and could also persist, even with lower probability, in the secondary effluent. Figure 3 shows the monthly variation of their occurrence in raw sewage and secondary effluent during the sampling period. For raw sewage, the positive ratio was 3/3 in December 2010 and March, April, and May 2011, while 2/3 in the other months. For the secondary effluent, the positive ratio was 2/3 in November 2010 and May 2011, followed by 1/3 in December 2010 and March 2011. The frequent occurrence of HFMD viral pathogens in the secondary effluent (before disinfection) implies the high stability of HFMD pathogens during biological treatment in the WWTP. If the effluent is not effectively disinfected before being discharged into receiving water (as sometimes happens in China), the infection risk cannot be ignored.

### Phylogenetic analysis of HFMD pathogens

**Fig. 4:**
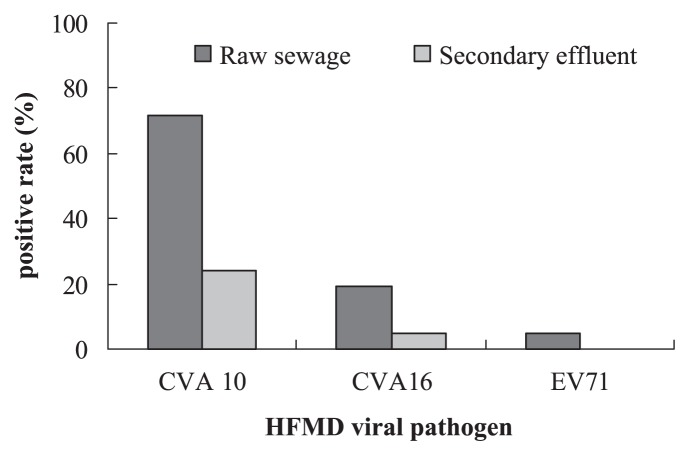
Positive ratio of three HFMD viruses in raw sewage and secondary effluent samples from a WWTP in Xi’an, China.

Figure 4 shows the occurrence of HFMD viruses in the collected water samples. For raw sewage, CVA10 was detected from 71% (15 of 21) of the samples, while CVA16 and EV71 were detected from 19% (4 of 21) and 5% (1 of 21) of the collected samples, respectively. One sewage sample (collected on May 5th) showed the coexistence of CVA10 and CVA16, and another sample (collected on November 23rd) showed the coexistence of CAV10 and EV71. For the secondary effluent, CVA10 was detected from 24% (5 of 21) of the samples, and CVA16 was detected from 5% (1 of 21) of the samples, while EV71 was not detected. CVA10 could thus be suspected as the main HFMD virus occurring in domestic wastewater in the study area, at least during the investigation period. The nucleotide identity between each HFMD virus isolated in this study and HFMD strains previously isolated in China was found to be about 97%, 95%, and 90% for CVA10, CVA16 and EV71, respectively. At the amino acid level, the identity ranged from 92% to 99%. These comparisons suggest that HFMD viral strains isolated in this study were genetically close to those previously reported in China.

**Fig. 5:**
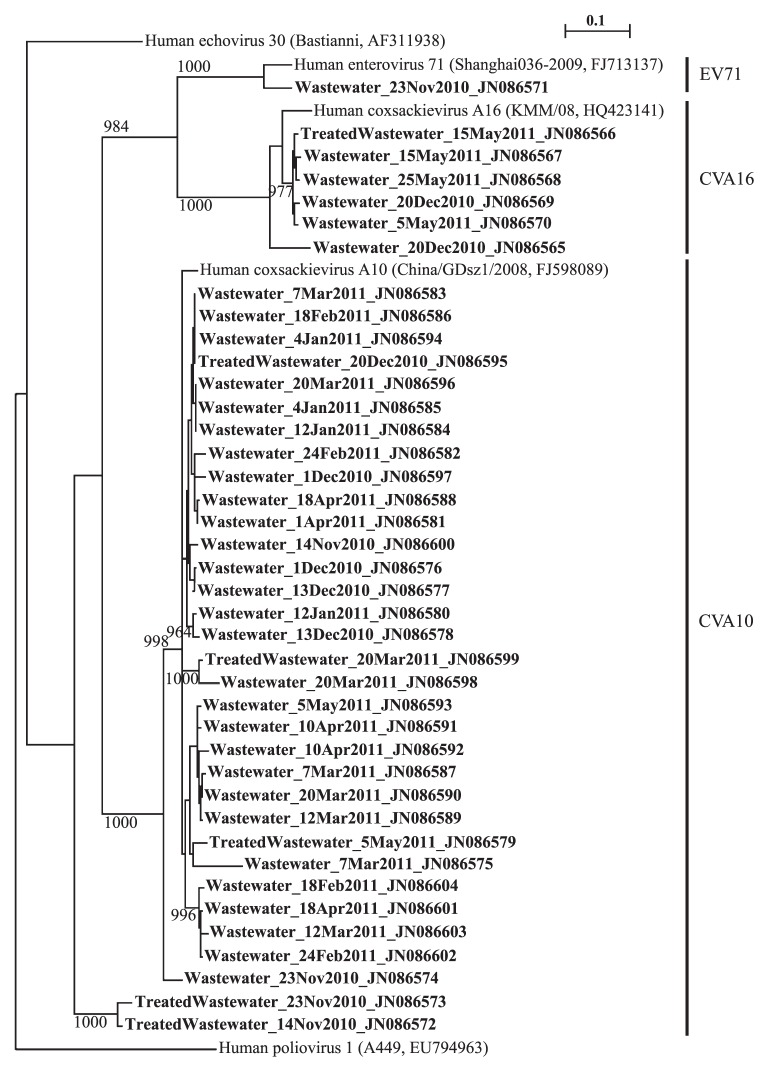
Phylogenetic tree constructed by the neighbor-joining method with 1,000 bootstrap replicates using ClustalX software (version 2.0.10). *Poliovirus type 1* and *Echovirus type 30* (*Enteroviruses B*) were used as outgroups. Only bootstrap values higher than 950 are displayed. The scale bar represents the number of substitutions per site. Bold type indicates sequences originating from HFMD viral pathogen obtained in this study. HFMD pathogens having the highest sequence identity with acquired gene sequences are indicated by right bars. The nucleotide sequences of HFMD viral pathogens determined in this study were deposited in the National Center for Biotechnology Information under accession numbers JN086565 to JN086604.

Overall, 40 sequences were obtained from wastewater samples so a phylogenetic tree, as shown in Fig. 5, could be constructed using the neighbor-joining method. Bootstrap analysis was performed by resampling the data sets 1,000 times. A single sequence was obtained from four sewage samples (collected on November 14th, April 1st, and May 15th and 25th, respectively), and multiple sequences were obtained from a single sample in more than half of all sewage samples (14 of 21). The sequences originating from CVA10 were highly diverse with shared nucleotide identity from 94% to 99%. The high mutation rate in the viral RNA genome is mainly owing to the lack of a proofreading function of RNA-dependent RNA polymerase during replication in human cells (17), implying that this serotype replicates frequently among the populations who have excreted sewage into the WWTP.

The frequent detection of diverse CVA10 is not expected because CVA10 has been a relatively minor pathogen for causing HFMD, although an outbreak of HFMD caused by CVA10 did occur in Shandong Province in China (26). Since the same level of detection limit (10^1^ copies/1st PCR reaction) was obtained for all three HFMD viral pathogens, as indicted in Fig. 2B, 2C and 2D, the frequent detection of CVA10 may not be attributable to different positive rates among serotypes. As can be seen from Fig. 5, diverse CVA10 strains were observed even in the non-epidemic season (January and February in general). It is postulated that asymptomatic infections largely occur in the investigation period, which is common in enterovirus infections (24). A recent report showed that a lack of correlation between the intestinal virus titer and enteric pathology was observed in murine norovirus infections (11). This study implies that asymptomatic patients could excrete a larger amount of replicated virions than symptomatic patients, although norovirus belongs to a totally different viral family from enteroviruses; however, asymptomatic patients as reservoirs and sources of enteric viruses in populations (5) should be highlighted to understand the environmental epidemiology of HFMD.

A more accurate description of different genotypes should be determined from the VP1 gene (18) in order to understand the characteristics of the epidemiological mechanism and genetic evolution of CVA10. Nevertheless, the primer set established in this study (targeting VP4 to VP2 region) could be useful for screening all three HFMD pathogens (EV71, CVA10 and CVA16) from wastewater samples. Although the existence of HFMD viruses in domestic wastewater may indicate the possibility of disease transmission by water media because the treated effluent from the WWTP, if not sufficiently disinfected, may finally enter a water body, the direct infectivity of HFMD pathogens in sewage and treated effluent is still unknown. Such a topic may need further investigation.

## Conclusion

The semi-nested RT-PCR developed in this study can provide a tool for rapid and sensitive detection of HFMD viral pathogens from wastewater samples. The simultaneous detection of three HFMD pathogens, EV71, CVA16 and CVA10, was achieved with the specific primer set targeting the conservative gene regions from 5′UTR to VP2. The high positive rate in raw sewage (86%) and treated wastewater (29%) indicated that HFMD viral pathogens were highly prevalent in the investigation area. CVA10 might be the main HFMD pathogen in the investigation area, as was revealed by sequence determination and phylogenetic analysis of the acquired viral gene. Further studies are needed on the infectivity of HFMD viral pathogens and the health risks posed by these pathogens in water.
